# Late-onset alopecia areata: descriptive analysis of 30 cases*

**DOI:** 10.1590/abd1806-4841.20165184

**Published:** 2016

**Authors:** Rosana Lazzarini, Camila Bilac Oliari, Ana Luisa Nasser Erthal

**Affiliations:** 1 Irmandade da Santa Casa de Misericórdia de São Paulo – São Paulo (SP), Brazil

**Keywords:** Alopecia areata, Clinical diagnosis, Descriptive epidemiology

## Abstract

Alopecia areata is an autoimmune disease characterized by non-scaring hair loss. The onset in over 50-year-old patients is rare and has barely been studied. Cases of this disease have been retrospectively analyzed – according to clinical forms, extension, and associated diseases – to assess alopecia areata characteristics in a group of patients whose disease onset was after the age of 50. 30 patients were studied; a few of them presented with autoimmune-related diseases or family history. The disease onset after the age of 50 seems to have different characteristics from those found in young people.

Alopecia areata (AA) is a common disease whose clinical manifestations range from mild lesions (i.e. plaques) to total hair loss (universal). Of unknown etiology, AA is autoimmune and genetic-related, affecting people of all ages, but mostly under 30-yearold patients. Only 20% of cases started in patients over 40 years old, and is very unusual after 50.^1^ The manifestations of the autoimmune lesions depend on age, gender, as well as clinical and lab findings, which are different from those found in young patients.^2^ This study aims to assess the epidemiological and clinical characteristics of a group whose disease onset occurred after 50 years of age.

A retrospective observational study of AA was conducted in patients treated between January 2000 and December 2014. The following criteria were analyzed: gender, age, clinical forms (plaques, ophiasis, ophiasis inversus, total, universal, and diffuse), nail changes, family history, associated diseases (atopic dermatitis, vitiligo, psoriasis, hypertension (AH), diabetes, anemia, rheumatoid arthritis, lupus erythematosus, inflammatory bowel disease, and atopy) and Down syndrome. The extension has been described according to the National Alopecia Areata Foundation criteria for loosing hair area as: S1 (<25%); S2 (26-50%); S3 (51-75%); S4 (76-99%) and S5 (100%).^3^ Laboratory tests included complete blood count, fasting blood glucose, urine, thyroid-stimulating hormone (TSH), free thyroxine (FT4), thyroglobulin antibodies (anti-TGL), and anti-thyroid peroxidase antibodies (anti-TPO). The data obtained were compared to the literature.

Of the 412 patients with AA treated during the period, 30 (7.3%) were included in this study--19 females (63%) and 11 males (37%). The onset age ranged from 50-82 years; median age was 61.3 years. 2 (6.7%) had a family history of AA; 19 (64%) had plaques with alopecia; 7 (23%) had universal lesions; 3 (10%) had ophiasis; and 1 (3%) had total lesions. Regarding extension, 16 were classified as S1 (54%), six as S2 (20%), one as S3 (3%), and seven as S5 (23%). No patient was classified as S4.

20 patients (66.7%) had at least one of the associated systemic diseases, seven (23%) of which were AH, and three (10%) were diabetes melitus. There was also one case of rheumatoid arthritis and one of hepatitis C (3% each). No cases of Down syndrome, inflammatory bowel disease, or systemic lupus erythematosus were reported. Two cases were systemic-medication-related: one anti-TNF and one ribavirin and interferon. Of the 30 patients, 21 (70%) had their thyroid functions tested. Five of them (24%) were positive for antithyroid antibodies, and seven (23%) had a previous diagnosis of septic hypothyroidism under treatment. Nails were affected in three patients (10%)--one with dystrophy in all 20 nails, one with cupuliform depressions, and one with longitudinal grooves.

The onset in patients over 50 years of age occurred in 30 participants (7.3%). A 2014 study with 513 patients showed that 18.7% of patients were over 50 years old when the disease first appeared.^4^ This difference may be related to the type of study, as the first refers to a spontaneous demand, while the second refers to active search. As for gender, this group had more females than males (63%/37%). In two epidemiological studies, men under 50 years of age were more affected, while women over 50 were slightly more affected, a fact confirmed in this group.^1,4^ The median onset age of the disease was 65.7 years (50-82 years), while another similar study reported 57 years (50-78 years).^1^ A 82-year-old patient skewed the figure in that study. In this study, only two cases (6.7%) showed family history, while the literature shows that family cases are present in 10-25% of AA patients.^5^ The difference may be related to different factors, such as a missing diagnosis of the disease in the family.

Regarding the extension, most cases were classified as S1 and in plaques. This is consistent with the literature, as S1 is the most common form of AA, regardless of age.^2^ However, this study sets apart from the others as it reported more patients with the universal form (7/23%).^1^ This difference may be related to the fact that clinics treat more patients with the more severe forms of the disease. Nail changes were present in three cases (10%). According to the literature, the most common changes are cupuliform depressions, which were absent in this study, probably due to the small sample size. The coexistence of other diseases occurred in 20 cases (66.7%), more than that observed by Wu *et al.*, possibly due to the type of population studied.^1^ Among the associated diseases, thyroid disease was observed in 7 patients (35%). The incidence ranges from 8-28% of cases in different studies, regardless of age.^2^ A similar study found even less frequency (2.7%)^1^. In AR and hepatitis C patients, the disease was directly related to the treatment, in which hair regrowth occurred after medication withdrawal. This etiology should be taken into account when using drugs that affect the immune response.^6,7^

Although this is a retrospective study with a small number of patients, peculiar characteristics of the disease have been observed, such as reduced association with autoimmune diseases and family cases. However, more studies are required to define the characteristics of AA in this group.
